# Lessons from the Women and Gender Constituency: Interrogating Civil Society Strategies for Organising in the UNFCCC

**DOI:** 10.1007/s10767-023-09448-z

**Published:** 2023-04-06

**Authors:** Joanna Flavell

**Affiliations:** Department of International Relations, London School of Economics and Political Science, Houghton Street, WC2A 2AE London, UK

**Keywords:** WGC, UNFCCC, Civil society, Feminist activism, Sustainability politics

## Abstract

While scholarship on the topic of gender and the environment is steadily growing, little is known about the challenges faced and successes seen by women and gender NGOs operating as a central part of environment-focused civil society. In this paper, I offer such an analysis, examining the political strategies—rhetorical and procedural—mobilised by the Women and Gender Constituency (WGC) in the United Nations Framework Convention on Climate Change (UNFCCC). I argue that the WGC has seen lots of success in mobilising arguments that foreground women’s vulnerability to the effects of climate change. But at the same time, the constituency has seen far more resistance to more intersectional feminist arguments that interrogate the role of masculinised discursive power in shaping climate politics. This is at least in part a result of a wider structure of civil society that pigeonholes different identities (e.g. gender, youth, indigenous peoples) in a way that separates their deeply interconnected struggles. Understanding this structural barrier, or dark side of civil society, is crucial to envisioning a more fruitful integration of civil society in sustainability politics.

## Introduction

In a 2011 paper, gender and environmental scholar Seema Arora-Jonsson argues that “dual themes” feature in the existing literature on gender and climate change: women as vulnerable or women as virtuous. The presentation of women as vulnerable entails that they are disproportionately affected by climate change either because of their disproportionate poverty (Hemmati & Röhr, 2007; cited in Arora-Jonsson, 2011) or cultural and gender norms in many contexts that result in higher mortality rates in natural disasters (Brody et al., 2008, cited in Arora-Jonsson, 2011). Alternatively, when presented as virtuous, women are seen as more risk-adverse, better prepared for behavioural change and more likely to support drastic policies and measures on climate change than their male counterparts (Brody et al., 2008; cited in Arora-Jonsson, 2011; see also McCright & Dunlap, 2011 for a discussion on climate denial among white conservative American men). Arora-Jonsson (2011) points out that this is problematic because framings of gender that focus on a single-axis of identity (such as women’s class or poverty) can do political damage by reinforcing stereotypes. This can potentially lead to an increase in women’s household, or social reproductive, labour, such as responsibility for recycling or mending clothes in the name of increasing sustainable practices. Furthermore, focusing on women’s vulnerability or virtuousness can deflect political attention from other kinds of inequalities, for example as those in decision-making in political institutions focused on sustainability, such as the UNFCCC.

These kinds of criticisms of the framing of the “gender and climate change” issue employed by feminist activists are common in literature on gender and climate politics. For example, feminist environmental scholar Bernadette Resurrección explores how women-environmental linkages remain “seductive and influential” (2013, p. 33). Simplified depictions of women as atomised individuals with fixed attributes, found in policy translations of Women Environment and Development [is this referring to a specific organisation or document?], are entirely dissociated from wider relationships of power and so can be counterproductive for women insofar as they reify traditional gender roles. Yet images that signify a victim status of rural women in the Global South have proven to be a successful strategic entry point for feminist sustainability advocacy. Pointing specifically to the gender experts who were (and in many cases still are) active in the UNFCCC under the umbrella of the Women and Gender Constituency (WGC), Resurrección demonstrates how the iconography of women as climate victims has drawn significant attention to their cause from the climate negotiating tables and thus remains a persistent rhetorical strategy in feminist climate advocacy. This presents a clear strategic dilemma for feminist civil society organisations such as the WGC: how can feminists make collective political demands without invoking a homogenised framing of gender, centred on women as vulnerable victims? It is this question that motivates my research in this paper.

The WGC is comprised of 34 NGOs that observe the annual UN climate conferences as one constituency within civil society (WGC, 2022). The WGC is the primary platform for observer organisations working to ensure women’s rights and gender justice within the UNFCCC framework and, thus, is an important focus for understanding the role of feminist civil society in sustainability politics. Yet little is known about the institutional conditions the WGC operates under, efforts by its members to advance feminist arguments in the UNFCCC, or the political strategies they have mobilised in order to make them. Scholars who have levelled some criticism at the WGC, or feminist environmental civil society actors more broadly, have tended to comment on the problematic results of decisions made in the face of dilemmas and difficulties (c.f. MacGregor, 2006; Arora-Jonsson, 2011; Resurrección, 2013; Sasser, 2017). This criticism is necessary, important and valid. But there is also a need for empirically rooted research that takes these kinds of criticisms and examines the processes and strategies that have succeeded in inserting a gender perspective into institutions of global climate governance.

The purpose of this article, then, is to examine the political strategies mobilised by the WGC in order to understand what the implications of the challenges and successes of the WGC might mean for civil society involvement in climate and sustainability politics more generally. Through fieldwork completed between 2017 and 2018 coupled with desk research thereafter, I combine an analysis of elite interviews with those working in the UNFCCC space, both as gender activists and negotiators on gender issues, with an analysis of hundreds of documents relating to gender in the UNFCCC to investigate the political strategies that have been mobilised by the WGC. Based on my analysis, I split these strategies into two broad approaches: rhetorical strategies (which discursively frame the connection between gender and climate change) and procedural strategies (the means of advocating for such rhetorical strategies within the UNFCCC). The distinction between these approaches is an organisational one, though they are very closely related. This research demonstrates that the WGC has experienced great success in mobilising arguments that foreground women’s vulnerability to the effects of climate change (in contrast to men’s presumed invulnerability), but there has been resistance to its arguments that interrogate the discursive workings of masculinised power in shaping environmental politics. This is, I argue, at least in part a result of the nature and structure of civil society participation within the UNFCCC, and indeed in sustainability politics more broadly; an attempt to split civil society into neat and well-defined constituencies that ultimately pigeonhole different identities (e.g. gender, youth, indigenous peoples) in a way that separates their deeply interconnected struggles. Understanding this structural barrier is crucial to envisioning a more fruitful integration of civil society in the politics of sustainability.

## Feminist Activism and Civil Society

As demonstrated above, there is a clear tension between the strategies for civil society engagement in sustainability politics. This tension is not unique to the gender and environment literature but is found across various forms of activism and advocacy in civil society and is expressed within a debate concerning the choice that social movements must make between working as radical outsiders or pragmatic insiders. In other words, is it more effective to work within existing political institutions and agitate for reform, or to remain outside of those dominant political systems with an aim of more fundamental structural change in how society works? This debate is found within writing on New Social Movements (c.f. Doherty, 1992, 2006; Diani, 1992; Ciplet, 2014) and also a small body of feminist literature that examines the political strategy of gender networks (c.f. Alber, 2009; Bretherton, 1998, 2003; Röhr, 2009). Bretherton has theorised the dilemma, stating that the “principal division, in terms of women’s organising, lies in the choice between insider and outsider strategies” (Bretherton, 2003, p. 108). Meyer and Prügl sum this up neatly in reference to women’s international NGOs:

Some women’s international NGOs (WINGOs), particularly the older ones, are more formally organised and seek to influence more directly the agendas of multilateral institutions. Other WINGOs, particularly those founded since the 1970’s eschew this formality and its related hierarchy and bureaucracy, preferring instead to mobilise and organise women at the grassroots and national levels around global feminist issues like development, population, women’s human rights, violence against women, environment and so on (Meyer & Prügl, 1999, p. 8).

The WGC exhibits predominantly “insider” characteristics, employing “an innovative lobbying strategy that has vastly increased the participation of non-governmental organisations (NGOs) at recent UN conferences” (Higer, 1999, p. 130). But these strategies are supplemented by mobilising women outside of official processes by, for example disseminating insider information to women’s networks at local levels to inform their political activism. This kind of dual process characterises much of women’s participation in UN conferences: the insider approach has allowed the women’s movement to become a highly visible player at the policy table, while outsider approaches have allowed it to articulate an alternative policy framework to existing international institutions (Higer, 1999). Yet Bretherton (2003) argues that while women’s movements have made considerable gains in their advocacy efforts, their strategy of engagement can only advance arguments within existing structures, not change them. The dilemma is that networked women must learn to work within existing processes of global governance, which Bretherton argues inhibits actions intended to undermine dominant norms and practices.

There are many benefits of working inside dominant political structures and institutions such as the UNFCCC. A strategy of engagement, developing centralised, hierarchical organisational structures and levels of expertise enables gender advocates to contribute to, and disseminate, information in order to challenge the dominant narratives of climate change (Bretherton, 1998). And as Hemmati and Röhr point out, “if women’s organisations are not actively involved, gender and women’s aspects will not be addressed” (2007, p. 6). In other words, getting the word on the page requires a seat at the table. But that seat at the table often requires the mobilisation of limited framings of identity.

Participation by societal actors is implicit in the concept of global governance. Social movements, including the women’s movement, are encouraged to participate in global governance, hence the inclusion of civil society in the UN system, including the UNFCCC (Bäckstrand et al., 2017). Most organisations proclaim their eagerness to listen to women’s caucuses (or constituencies in the case of climate change). In return, there is an expectation of access and influence. Through the UN, NGOs have more access to the policy-making process than ever, and for many NGOs, this level of participation is seen as a positive step in gaining more influence around the negotiating table: they are necessary partners who provide significant contributions to policy texts. But these NGOs may have overestimated their influence since they also operate in a global system of push and pull from transnational corporations and global finance (Stienstra, 1999). Understanding this dilemma is important because “divisions are not trivial; choice of strategy and related ideological position are important determinants of cohesiveness in women’s movements” (Bretherton, 2003, p. 109). I argue that understanding these dilemmas, and the means by which civil society overcomes them (or not), is crucial for envisioning a more fruitful civil society engagement in global sustainability politics.

## Rhetorical Strategies Mobilised by the WGC

Based on my analysis, I categorise the rhetorical strategies, or the arguments made to advance specific framings of the issue, mobilised by the WGC fall into three main themes: gender equality and empowering women; foregrounding women’s vulnerability and intersectionality in baby steps.

### Gender Equality and Empowerment of Women

There has been a lot of success in mobilising rhetoric around empowering women in the name of gender equality in the UNFCCC. For example, Decision 36/CP.7, the very first gender mandate under the UNFCCC, recognised the importance of women’s empowerment and full participation in climate action (UNFCCC, 2001, p. 1). Similarly, the Paris Agreement acknowledged the importance of the empowerment of women (UNFCCC, 2015, p. 2). The issue of women’s empowerment is commonly understood as necessitating greater participation by women in the UNFCCC negotiating processes. As one practical means of ensuring this, particularly involving developing countries, the Global Gender and Climate Alliance (GGCA)—formed of the Women’s Environment and Development Organisation (WEDO), the United Nations Development Programme (UNDP) and the International Union for Conservation of Nature (IUCN) in 2009—partnered with the government of Finland to launch the Women’s Delegate Fund (WDF). Over the years, the WDF has drawn support from several other donors including the governments of Australia, Canada, France, Iceland, the Netherlands, Scotland, Sweden and Switzerland (WEDO, 2021). Administered by WEDO, the fund provides travel support for women from least-developed countries (LDCs) to attend the UN climate change Conference of the Parties (COP).

Campaigners for gender equality and women’s empowerment do not only focus on increasing the number of women delegates within the UNFCCC itself. They are also want to increase women’s participation in various adaptation and mitigation efforts, particularly in the Global South. Typically, these kinds of strategies will work to promote women’s participation in, and allow women to benefit from, green climate solutions such as technology transfer. A factsheet published by ENERGIA^1^ and WEDO in 2010 called “Recommendations for Climate Negotiators on Energy Technologies and Gender Equity” is a good example of this. The factsheet stated that:

The technology transfer, capacity building and financing provisions of climate agreements and response plans should be inclusive and equitable so that both women and men can have access to, and benefit from, the development and transfer of new energy technologies (ENERGIA & WEDO, 2010, p. 1).

Underpinning such arguments is the idea that women should be able to take advantage of a Green Economy type approach to climate action, by accessing equal business opportunities to improve their opportunities for economic empowerment. In this way:

The role of women as energy providers can be transformed into suitable micro-enterprises if they can manage fuel wood or oil seed plantations, dispense kerosene or liquified petroleum gas (LPG), assemble solar panels, build cook stoves and brick kilns, and even manage electricity distribution and bill collection (ENERGIA & WEDO, 2010, p. 1).

As discussed above, these strategies have been, on paper at least, successful in influencing policy. But as Anniete Cohn-Lois, WDF Delegate from the Dominican Republic, warned, “at the national level we speak a lot about the importance of women empowerment, but it’s not the same thing as actually implementing policies to achieve progress on this” (Burns & Andre, 2014, p. 6). This suggests that the term “women’s empowerment” is a handy phrase to draw upon but is rarely followed up by meaningful political action (see Buckingham & Kulcur, 2009). I turn to strategies to overcome this issue below.

### Foregrounding Women’s Vulnerability

So, this was for me, it really was the starting point and just because we got attention only by evidencing women as the most vulnerable. So, what we have to do, my experience was, to get to end with gender in these processes, or women into the process, we had to take this approach as an entry point on women are the most vulnerable. And so, for that reason, and I remember it very well in Bali, you’ll have to look for the dates or COP numbers, but in Bali that was for me the first conference where much more focus was put on adaptation. Beforehand it was much more on mitigation, so it was a shift in issues because of pressure from development corporations, or organisations, working in these areas (Röhr, 2017, interview).

COP13 Bali in 2007 represented a pivotal moment in gender advocacy at the UNFCCC. Members of the Constituency made the decision to speak to the wider shift in UNFCCC discourse from mitigation towards adaptation, which resulted in the pursuit of a rhetorical strategy that foregrounded women as vulnerable victims of climate change. Prior to COP13 Bali, the network of gender advocates active in the UNFCCC had avoided discussing the specificity of women located in the Global South, instead favouring an approach that focused on the roles of masculinity and male power in shaping climate discourse. For example, at an informal meeting of gender-focused climate activists at COP9 Milan in 2003, the attendees noted that “the process tends to be driven by a masculine view of the problem and its solutions” (LIFE et al., 2003, p. 1).

The WGC shifted in 2007 from what can be described as a strategy that foregrounded issues of male power and masculinity to one that rhetorically equates gender with women’s vulnerability in the Global South, mirroring the wider shift in the UNFCCC. More broadly within climate discourse, the political conversation shifted from how to halt climate change (mitigation) towards how to deal with the most harmful effects of climate change (adaptation). The WGC responded, shifting to a strategy of foregrounding the lived experiences of vulnerable women in the Global South. For example, several high-level statements and plenary interventions from the time began to highlight women’s vulnerability to the most severe effects of climate change (e.g. Aguilar, 2007; Brundtland, 2007; Zeitlin, 2007). They saw this as a means of influencing policy and had great success in doing so. The 2010 Cancun Agreements, for example make special reference to “those segments of the population that are already vulnerable owing to geography, gender, age, indigenous or minority status, or disability” (UNFCCC, 2010, p. 2).

There have been some practical benefits for the WGC working under such a framework. The move allowed the network to increase its legitimacy as a group working inside the UNFCCC. It also provided the tools to help make the links between gender and climate change clear to Party negotiators:

Initial attempts to link gender and climate change may seem rather *far-fetched* especially for the sceptic. However, analysing the issues from a poverty, vulnerability, environmental resource management, equity and sustainability angle the links become *inherently obvious* (ENDA Tiers Monde, 2002, p. 7, my emphasis).

The WGC makes these links in a variety of ways, for example in a series of case studies published between 2008 and 2009 that highlight issues in individual Global South countries including the Philippines, Bangladesh, Senegal and Ghana (Alam et al., 2008; Gueye, 2008; Henrich Böll Stiftung & WEDO, 2009; Mensah-Kutin, 2008). In one case study, Yacine Diagne Gueye of Environmental Development Action in the Third World (EBDA) gives an overview of climate change in Senegal, drawing attention to the implications for women’s livelihoods, security and gender equality. She writes that women in Senegal face several difficulties including access to water as a result of a 35% decrease in rainfall, concerns over energy and impact on the fishing industry, in which than 90% of women are involved (Gueye, 2008).

The purpose of these case study examples is to highlight the main issues faced by women across the developing world and to provide entry points for feminist advocacy in the UNFCCC. However a strategy foregrounding women’s vulnerability to the effects of climate change is not without its challenges. While these roles represent an empirical reality for many of the world’s women, they are not a universal female experience. Yet extrapolating women’s vulnerability as an answer to the gender and climate change question remains an all-too-common reaction. As MacGregor (2017) points out “when pressed to explain what gender has to do with climate change, it is tempting to point to impacts and vulnerabilities” (p. 16). In making this claim, MacGregor points to the answer given by Mary Robinson, former President of Ireland and active advocate for gender equality in the UNFCCC, when asked what gender equality has to do with climate change:

Oh, there are huge gender impacts of climate, huge impacts. If you undermine poor livelihoods, who has to pick up the pieces? Who has to put food on the table? Who has to go further in drought for firewood? … Those who are trying to adapt and be resilient, the vast majority of farmers in the developed world, are women. So, it is usually important that the gender dimensions are recognized, both the fact that women are mor vulnerable, because even in natural disasters they are … more likely to die than men – 14 times, because they care about their children and try to hug them, they can’t run very fast in long clothes – a whole variety of reasons’ (Robinson 2015 cited in MacGregor, 2017, p. 16).

These kinds of narratives should be credited for their role in getting the issue onto the policy agenda. However, a disproportionate focus on the material impacts on and differential vulnerabilities of women compared with men has limited the political scope of the issue and diverted attention away from issues of structural imbalances of power, including patriarchal power (MacGregor, 2009, 2017), ultimately universalising the lived experience of all women based on their disproportionate vulnerability to climate change compared to men’s presumed invulnerability.

The choice made by the WGC to focus arguments on this specific experience, then, was a pragmatically strategic one. Howell and Mulligan (2005, p. 5) suggest that women are typically excluded from male-dominated politics (both climate politics and more broadly) and so find routes into participating in civil society at local levels. The case studies presented by the WGC are one concrete means of bringing those grassroots women into male-dominated political spaces such as the UNFCCC. Members of the WGC are aware of the potentially harmful effects of strategies that foreground women’s vulnerability and know that more nuanced arguments are needed:

We [US communities of colour] know that solutions look different for our communities and there is no one-size fits all, (and) recognise that we must resist in the different ways that we can (Managaliman, 2016; cited in Acha, 2016, p. 13).

But, as Ghani frankly put it in an interview: “you could be a raging feminist, but you never bring that into your conversations or the work that you are doing. There’s no space. It’s just not possible”. Similarly, Röhr distinctly remembered trying to bring up masculinity during an intervention to much resistance from both party negotiators and civil society. This is just one example of the dilemmas facing the WGC and its members as feminist activists working inside the system.

### Intersectionality in Baby Steps

It is clear from my research that the WGC and its members are committed to principles of intersectional feminism which allow discussions that highlight (rather than play down) multiple axes of difference (see Kaijser & Kronsell, 2014). The concept of intersectionality posits that inequality, oppression and marginalisation do not happen in a gendered vacuum, but are made up of multiple and intersecting power relations that shape identity politics such as race, class, gender, ability, ethnicity and sexuality, to name a few (Crenshaw, 1991). These more nuanced discussions are particularly explicit in internal training events (c.f. Burns, 2013; Reyes & Burns, 2013; Röhr, 2013) but because they rarely make it into policy documents they can easily be overlooked. However, there was a moment of reflection within the Constituency in 2013 about the use of problematic rhetorical strategies to get women’s issues on the negotiating table, for example gender being used as synonymous with women; the terms gender/women implying vulnerable or victim and women as poor or altruistic stewards of the environment. The presenters called for a more embedded intersectional approach to gender advocacy that moves away from these limiting constructions of gender, instead drawing attention to the specificity of women located in different geographical locations, as well as where they might sit on different axes of marginalisation and oppression (c.f. Reyes & Burns, 2013). A series of internal capacity-building events stressed that intersectionality was not only relevant for research but for policy-making too. They also encouraged members of the WGC to be aware of the politics of knowledge production, to be more specific when talking about women and gender and to make clear the different situations of women in different contexts. As a result, the Constituency agreed that its members should share expertise with the aim of coming up with a common understanding of gender issues (Burns, 2013) and that they should pursue a two-track strategy of continuing to lobby for the inclusion of gender language in UNFCCC texts while also continuing the internal discussion about non-essentialist and intersectional approaches and building capacity within the Constituency (Röhr, 2013).

Since then, there has been an increase in explicit articulations of intersectional principles, with the term being used more commonly in the Constituency’s demands. For example, in an eDiscussion on Climate and Environmental Justice held in 2016, Maria Alejandra Rodriguea Acha, Co-Executive Director at FRIDA - The Young Feminist Fund, the co-founder and former co-coordinator of TierrActiva Perú, claimed that “groups are increasingly rejecting single-issue campaigns and demands and recognising the intersectionality of diverse movements and struggles” (Acha, 2016, p. 13). To this end, the term intersectionality was explicitly mentioned in the Gender and Action Plan (GAP) negotiations and was incorporated into an informal summary of a workshop, to stress that implementation of climate solutions must “highlight intersectionality and broader social contexts as part of gender-assessments” (UNFCCC, 2017a, p. 5).

Despite these small wins, the Constituency is aware that it faces huge challenges in gender rhetoric at the official agreement and policy level because:

while gender issues have now a higher profile in the UNFCCC, a normative understanding of the link between gender equality and climate action, and the role of the gender mainstreaming strategy is still very limited (Burns & Lee, 2015, p. 18).

Burns and Lee go on to suggest that, within the UNFCCC, challenges in advocating for the inclusion of gender concerns are partly down to limited capacity and resources. But critically there is gap in knowledge about the social dimensions of climate change issues among the majority of those engaging in climate policy from a scientific, technical and financial background. As such, the WGC pursues intersectionality in “baby steps” or, as Morrow (2017) suggests, it favours incremental progress towards the end goal of a feminist-informed climate politics.

## Procedural Strategies

Rhetorical strategies can only be effective if they are coupled with procedural strategies, or the mode of engagement to advance arguments within the institution. The Constituency has developed a particular modus operandi over the years in order to “speak truth to power” (Röhr et al., 2009, p. 297). As I see it, this modus operandi is organised around three complementary activities: identifying entry points for gender aspects into the climate change debate; raising awareness and disseminating information; building women’s capacity and joint strategising.

### Identifying Entry Points for Gender Aspects into the Climate Change Debate

As a first point of reference for lobbying for gender issues to be integrated into global climate change policies, the WGC draws upon the links between gender equality, women’s rights and climate change that exist outwith the confines of the UNFCCC itself. UN declarations such as the Beijing Platform for Action (1995) are particularly significant because Parties to the UN have already committed to its mandate for gender mainstreaming. Similarly, the Convention on the Elimination of all Forms of Discrimination Against Women (CEDAW) has direct implications for climate change policy because it obligates Parties to the UN to take:

all appropriate measures to eliminate discrimination against women in rural areas in order to ensure, on a basis of equality of men and women, that they participate in and benefit from rural development (United Nations, 1979, p. 5).

More recently feminists have pointed to the Sustainable Development Goals (SDGs) and Agenda 2030. For example, during COP23 at an open negotiation for the Gender Action Plan (GAP), at which observers were admitted to the negotiating room to watch proceedings, one negotiator called for the GAP to make clear links to both Agenda 2030 and the SDGs, resulting in the preamble including the line “reaffirming the General Assembly resolution on the 2030 Agenda for Sustainable Development” (UNFCCC, 2017b).

The WGC also draws from existing UNFCCC documents such as the Paris Agreement. These links inform the WGC’s members when lobbying for the inclusion of specific language, such as loss and damage, human rights and gender equality. It gives the Constituency something concrete to pin arguments on while lobbying, as they have already been agreed and mandated by Parties. Emilia Reyes, negotiator for Mexico and human rights activist, summed this up by saying in an interview that “the biggest achievement of the GAP was including language on human rights for the first time under the gender agenda item”. She went on: “We don’t care about where it is, we just want the precedent”, in reference to gender equality appearing primarily in the preamble to the Paris Agreement. Therefore, it seems that the logic of much of the activist work around identifying entry points for gender aspects in the climate change debates is about getting specific words and phrases into documents, because once it is there they can act as precedents to be drawn upon in future negotiations.

### Raising Awareness and Disseminating Information

Efforts to get language on gender included in official policy are informed by a dedicated team within the WGC of primarily younger feminists, who design creative ways to raise awareness of gender and climate change issues at the COP. This includes the increasing use of actions, by which they mean strategies of disruption, as well as more traditional informational side events and exhibits. Typically, actions are designed to draw attention to recent or upcoming events, both at the COP and sometimes more broadly. One such example organised by the WGC called #MindTheGAP, performed at COP23 amidst the GAP negotiations in 2017, was designed to highlight the importance of the GAP and to reinforce demands for the negotiators. In a press release Hanna Gunnarsson, policy and communications officer for the WGC, wrote:

The Women and Gender Constituency views a comprehensive, targeted and resourced two-year gender action plan (GAP) as a critical outcome for COP23, in order to urgently advance gender-responsive and human-rights based climate policy and action (Gunnarsson, 2017).

The action itself was based around an aerobics class which included all the main priorities that the WGC argued were essential for a comprehensive GAP. In this spirit participants (myself included) and observers were urged to “reach for the money”, “lift the [gender-disaggregated] data” and to “fight for gender equality” (WECF, 2017a). More recently at COP26 Glasgow in 2021, in the wake of the COVID-19 pandemic, members of the WGC wore brightly coloured face masks reading “feminist climate justice” in a bid to call attention to, and start conversations about, the links between climate justice and gender equality (WEDO, 2021).

Actions do not happen in a vacuum; they are just one part of a package of activities designed to raise awareness and disseminate information. The WGC, along with other UNFCCC civil society members, also uses side events as a means of introducing potential agenda items for the negotiations, networking and connecting with people as well as promoting reports and research. Typically, events are in the form of panels with speakers on particular issues. For example at COP26 the WGC held side events on issues ranging from “uncovering justice gaps in just transitions” to “engaging citizens in urban climate action for inclusive just transition programs” (GenderCC, 2021). Previous research by Schroeder and Lovell (2009) has shown that about one quarter of attendees of side events are negotiators or government representatives. This means that side events are not a parallel world with little connection to the formal sessions, but can have a potential knock-on effect in the negotiations. They are an integral part of the UNFCCC process.

### Joint Strategizing

Creating an influential movement requires coordination, and one of the key strategies in representing the interests of women is to form networks or coalitions with like-minded COP participants and civil society organisations. The WGC organises throughout the year, hosting regular webinars and caucuses in order to build women’s capacity and to facilitate joint strategising among feminists. Morrow (2017) sees this as indicative of the Constituency’s strategy of practicing solidarity across issue areas. It is beneficial for NGOs to be a member of a network such as the WGC partly because it allows for coordinated national and international advocacy.

One means of joint strategising in the WGC is to invite representatives of other constituencies to the Gender Caucus. For example, during the COP events, the WGC hosts theme days at which women and feminists from other constituencies are invited to the caucus. During COP23, the WGC held several themed days including Young Feminist Day and Indigenous Women’s Day. Both were poignant, with the Young Feminists using poems to highlight issues faced by young women. On Indigenous Women’s Day, women from communities from around the world began with an invocation led by Tarcila Rivera Zea from Peru calling for an intersectional gender-just sustainable future. The event involved lighting candles and forming a circle sealed by holding hands for quiet reflection. More recently at COP26, members of the WGC joined an Indigenous People’s protest outside the conference centre in Glasgow calling attention to the ways in which carbon trading schemes can lead to projects such as biofuels and dams that cause the destruction of both Indigenous lands and lives (Lakhani, 2021).

Despite the divisions between the constituencies, building alliances between NGOs is imperative, not least in linking issues and allowing like-minded groups to push for human rights together. As such, submissions by various UNFCCC constituencies, particularly Environmental NGOs, WGC, Youth NGOs and Indigenous People’s Organisations often mention women’s rights, indigenous people’s rights and youth rights as well as climate justice more generally, in a bloc (see for example WGC, 2016, 2017). However, some members of the Constituency are wary about the kinds of alliances that are built and the coalitions that are formed. One interviewee, for example, said:

This morning I read in the advocacy list [an email list that is used for strategizing among the Constituency both during and beyond the COP] an advertisement for a side event that was weird. It was transformation of… of principles of Pope Francis, a book or pamphlet or something. Have you read it? It’s against gender, well it’s against reproductive rights of women, and we from the WGC promote it! I asked and they said, well yes but it’s [another member of the Constituency] who is on the podium. I said, well we should ask why they are on the podium! (Participant B, 2018, interview).

This speaks to what Yuval-Davis (1999) warned about the creation of epistemic communities—groups with similar and compatible values which can cut across differences in positioning and identity—whereby the divisions of who should be included, or where the boundaries of a community should be, are not always clear. Howell and Mulligan (2005) characterise civil society as:

a double-edged sword for feminists. It can provide a site for organising around feminist issues, for articulating counter-hegemonic discourses, for experimenting with alternative lifestyles and for envisioning other less sexist and more just worlds. With its organisations of self-support, community action and voluntary care, it can foster solidarity, promote mutual support and prioritise values of care, respect and equality. Yet it can also be an arena where gendered behaviours, norms and practices are acted out and reproduced (Howell & Mulligan, 2005, p. 6).

Joint strategising, then, is a complex process of identifying other civil society actors with similar values and goals. Joint strategising with other activists who are committed to the principles of climate justice, or principles which could be articulated together in a “chain of equivalence” (see Laclau & Mouffe, 1985; Mouffe, 2005), facilitates efforts not only for lobbying delegates in the creation of a cohesive message, but also for capacity-building activities which are of huge importance to the Constituency and for party delegates.

## Lessons from the WGC for Civil Society

We have a situation of inequality between women and men, and we have to respond to it. I want to change the situation, not to make the inequality less unequal, but to change the reasons for the inequality. That’s what we try to work on. *It’s hard. It’s hard* (Röhr, 2017, interview).

It is abundantly clear from watching and listening to activists who have worked to embed a gender perspective into global climate governance that it is *hard*. While the concerns about the political strategies of gender and environmental advocates outlined in the introduction of this paper are valid complaints with regard to the WGC, spending time with and engaging with the Constituency has shed light on the real-life challenges in the negotiating process and the difficult strategic decisions that they must make. Simplistic and universalising claims about women and their lives sit as uncomfortably with feminist activists in the UNFCCC as they do with feminist academics writing about them. In an interview Röhr commented that “from the very beginning I said I don’t want to use this argument—the most vulnerable—I want to go more about transformation, and the masculinities and so on”.

For the WGC, making arguments that foreground women’s vulnerability was an important strategic manoeuvre that was necessary in order to get gender concerns onto the negotiating table. In particular, women’s vulnerability in the face of climate change fits well with a wider discursive shift in the UNFCCC towards issues of adaptation as opposed to mitigation and with the kind of “special interest thinking” that underpins the UNFCCC. While a focus on women’s vulnerability has proven to be the most effective rhetorical strategy for influencing climate change negotiations, it is certainly not the only story told about women and their lives by the WGC.

While academic criticism is to be welcomed, it is often too abstracted from the concrete realities of feminist resistance within the UNFCCC. In discussing criticisms levelled at the WGC for not making enough progress on gender in the UNFCCC by researchers who are removed from the COP process, Röhr commented in an interview that “I think if you are writing about these processes then you don’t have to be here three years every time, but to get a feeling how it works is quite important”. This process of seeing how it is, watching, talking and listening to feminists trying to make collective political demands has been integral to my research. Doing so has fostered an appreciation of the challenges facing gender and environment advocates and, in this light, I find the successes of the Constituency to be remarkable. Morrow (2017) has also come to this conclusion after sustained engagement with the WGC. Gender equality is now reflected in a number of key UNFCCC decisions and mandates, including the Standing Agenda Item of Gender and Climate Change giving the issue both weight and prominence within the negotiations. More concretely, the actions of the Constituency have improved the lived experiences of many women, an achievement that should be celebrated by feminist environmental activists and academics alike. For example, through both the mentoring and financial support of the Constituency, Dorothee Lisenga from the Coalition of Female Leaders for the Environment and Sustainable Development (CFLEDD) has helped women in the Democratic Republic of the Congo (DRC) gain access to land and forest titles. Through dialogues on women’s inheritance rights between customary chiefs, local and indigenous women, this work has led to huge success in women’s access in the provinces of Ecuador and Maindombe of the DRC (see WECF, 2017b). Similarly, at least in part due to lobbying by members of the WGC, there has been an increased proportion of women in delegation parties from slightly over 20% in 1996 to almost 40% in 2017 (see UNFCCC, 2018).

It is clear from the success of the WGC that through engagement inside the UNFCCC, the conditions under which the Constituency operates today are very different from those that shaped its political strategy in the late 1990s and early 2000s. Early organising was concerned with gaining traction for social arguments in a political space that was only concerned about scientific, technological and economic arguments. Social concerns such as gender equality are now firmly on the agenda. The Constituency has done much to aid the shifting the agenda of the UNFCCC to a more socially just framework, yet still struggles to get multi-axis arguments about identity into important UNFCCC mandates.

One key structural barrier facing the WGC and hindering such multi-axis arguments about gender is the institutional structure through which civil society is invited to engage in the UNFCCC. Many of the Constituencies that represent different parts of civil society are based on specific identity markers—gender, indigeneity, youth—and so help sustain conceptual entities such as “woman” that are constituted through acts of exclusion or “othering” (Moore, 2008; see also Ahmed, 1998). In other words, the feminist struggle is separated from the Indigenous struggle, which is separated from the broader environmental struggle. The inclusion of civil society through constituencies is an attempt to level the playing field and ensure marginalised voices (such as women, indigenous peoples, young people and even the environment through ENGOs) are included in the process. But this can work to hinder progress. In effect, women, and the female body, are set up as a variant to the “normal” male (see also Buckingham & Kulcur, 2009). Men, after all, do not need to be represented through a special interest constituency; they are governed by different rules. Much has been written about the role and legitimacy of civil society in environmental governance (see, for example Biermann et al., 2012; Bäckstrand & Kuyper, 2017; Bäckstrand et al., 2017), but rarely is the basic concept of constituencies, envisioned in their current form, scrutinised or criticised. Indeed, the inclusion of civil society at United Nations Conference on Environmental and Development (or Rio 1992) is generally hailed as a positive step in widening the scope of the UN and expanding the political sphere beyond a space that is reserved for “statesmen” and lawmakers. But it is clear from this analysis that there is a dark side to the structure of constituencies that ultimately governs civil society’s involvement in processes of global climate governance, and indeed sustainability politics more broadly. The division of Constituencies in terms of identity markers is actually a hindrance to the full and successful participation of CSOs that advocate across multiple and intersecting lines of social inequalities.

Keeping the WGC in its own pigeonhole (while backgrounding gender and foregrounding women) forces its members to make political demands based on single-axis framings of identity. Pigeonholing civil society also serves to keep multi-axis identities atomised and separate from each other. Gender (meaning women) typically appears in a (short) list of social inequalities. Gender equality and the empowerment of women (an important rhetorical strategy for the WGC) is included in the preamble of the Paris Agreement in this way:

Acknowledging that climate change is a common concern of humankind, Parties should, when taking action to address climate change, respect, promote and consider their respective obligations on human rights, the right to health, the rights of indigenous peoples, local communities, migrants, children, persons with disabilities and people in vulnerable situations and the right to development, as well as gender equality, empowerment of women and intergenerational equity (UNFCCC, 2015, p. 2).

In this example, there is an implicit atomisation of experience in relation to the impacts of climate change. Experience (as an indigenous person, as a migrant, as a child, etc.) is defined as fundamentally individual and atomistic, subject to behavioural and attitudinal change to ensure their equality with men (on masculinist terms). Making collective and intersectional political demands in the UNFCCC fundamentally challenges the very fabric of the institution, which explains many of the challenges faced by the WGC. This is an important lesson for the study of civil society activities in sustainability politics because the next phase of feminist organising in the UNFCCC needs new kinds of strategies to overcome the challenges posed by intersecting domains of power that are features of the UNFCCC itself.

It is clear that the UNFCCC is shaped in ways that limit possibilities for successful gender advocacy, and civil society advocacy more broadly. This is not a new lesson for feminist environmental academics who make the case for intersectional arguments in global environmental governance. Indeed, MacGregor (2009, 2017) is one scholar who has been at the forefront of the call for “better answers” to the questions of gender and environment. Those better answers, MacGregor argues, would position environmental discourse as gendered, understand that there is a link between gender balance and policies advancing sustainability, and that gender justice is a necessary pre-requisite for any future sustainable world. These better answers would necessarily challenge the structural, cultural, disciplinary and interpersonal domains of power. It is not the case that such better answers do not exist, and members of the WGC hold many of those answers themselves. Importantly, however, what are missing are the strategies needed to embed those answers into political spaces of sustainability. This finding, this lesson from the WGC, should be a key area for future research on civil society in sustainability politics.

## Footnotes

1 ENERGIA is an international network of organisations focusing on sustainable energy and promoting the empowerment of woment. See https://www.energia.org/
